# *“It builds your confidence… you’ve done well”*: Healthcare workers’ experiences of participating in a low-dose, high-frequency training to improve newborn survival on the day of birth in Ghana

**DOI:** 10.12688/gatesopenres.12936.1

**Published:** 2019-05-22

**Authors:** Amos Asiedu, Allyson R. Nelson, Patricia P. Gomez, Hannah Tappis, Fred Effah, Chantelle Allen

**Affiliations:** 1 Jhpiego Ghana, Accra, Ghana; 2 Jhpiego Liberia, Monrovia, Liberia; 3 Jhpiego, Baltimore, MD, 21224, USA

**Keywords:** Low-dose high-frequency, skilled birth attendant, midwives, maternal and newborn care, in-service training, frontline health workers

## Abstract

**Background: **The majority of newborn deaths occur during the first week of life, and 25‒45% occur within the first 24 hours. A low-dose, high-frequency (LDHF) training approach was introduced in 40 hospitals in Ghana to improve newborn survival. The aim of this qualitative study was to explore healthcare workers’ experiences with the LDHF approach to in-service training.

**Methods: **A total of 20 in-depth interviews and nine focus group discussions were conducted in 2016 in three regions of Ghana with healthcare workers who participated in implementation of the LDHF training approach. In-depth interviews were conducted with 20 master mentors and peer practice coordinators; 51 practicing doctors, midwives and nurses participated in focus group discussions. Data were analyzed using a thematic analysis approach.

**Results: **Healthcare workers reflected on the differences between the LDHF approach and past learning experiences, highlighting how the skills-based team training approach, coupled with high-frequency practice and mobile mentoring, built their competency and confidence. As participants shared their experiences, they highlighted relationships established between Master Mentors and healthcare workers, and motivation stemming from pride in contributing to reductions in maternal and newborn deaths as critical factors in improving quality of care at participating health facilities.

**Conclusion: **This nested qualitative study documents experiences of healthcare workers and mentors involved in implementation of a multi-faceted intervention that effectively improved maternal and newborn care at health facilities in Ghana. The way the intervention was implemented created an environment conducive to learning within the hospital setting, thus providing an opportunity for professional growth and quality improvement for all staff working in the maternity ward.

## Introduction

### Background

Over the past 25 years, global newborn mortality rates have not decreased as dramatically as maternal and child mortality rates, despite an increase in skilled birth attendance and facility-based births ^1^. The majority of newborn deaths (75%) occur during the first week of life, and 25%‒45% occur within the first 24 hours ^2^. For the first time in history, intrapartum-related events are among the top three global causes of death of children under five years of age ^3^.

In 2015, Ghana ranked 53rd out of 225 countries with the highest infant mortality rates, even though 73% of births occurred in facilities with skilled birth attendants ^4^. Increasing the use of evidence-based interventions during labor and birth—infection prevention; monitoring the progress of labor with a partograph; and essential newborn care such as drying, provision of warmth, clean cord care, immediate initiation and exclusive breastfeeding, and resuscitation with bag and mask ventilation—could prevent many intrapartum stillbirths and newborn deaths in Ghana.

Improved effectiveness and scale of pre-service education and in-service capacity building for healthcare workers is necessary to implement the interventions to achieve better health outcomes. Recent reviews have highlighted substantial variation in the effectiveness of strategies to improve healthcare quality in low and middle income countries ^5^, and found there is little consensus on what distinguishes myriad interventions to support and improve healthcare worker performance (i.e. supervision, mentoring, coaching, quality improvement), nor on optimal approaches to delivering such interventions in different settings ^6, 7^. Evidence suggests that when used effectively, techniques such as frequent practice on anatomic models; simulation of the work environment; interactive discussion of case studies; problem-based learning; reminders; and the use of mobile media can bring about desired learning outcomes ^8^. Educational techniques that rely on passive transfer of information, on the other hand, such as lecture and reading, are less effective ^9, 10^.

### Program description

In 2014, Jhpiego introduced a “low-dose, high-frequency” (LDHF) training approach, in tandem with an ongoing quality improvement program in 40 district and regional hospitals across the Central, Western, and Upper West regions of Ghana ^11^. The objective was to transform the attitudes, skills, and knowledge of healthcare workers providing labor, birth, and postpartum care, in an effort to reduce preventable deaths.

The LDHF approach emphasizes skills competency through simulation and case-based learning, and appropriate spacing of training sessions with brief content delivery. By training midwives and doctors together and establishing a culture of learning at the health facility level, the LDHF approach reduces the time healthcare workers must spend away from work in offsite trainings, potentially increases opportunities for hands-on training, and could increase the number of healthcare workers trained at each site. 

**Figure 1.**
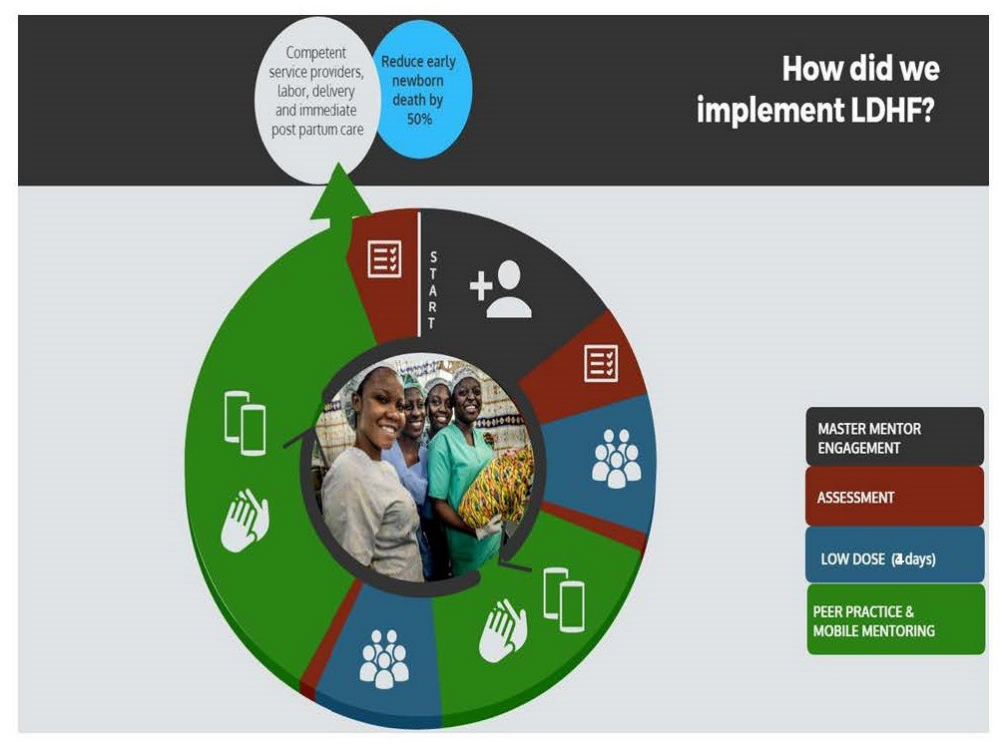
Low-dose, high-frequency training approach for newborn survival.

The Ghana LDHF training package had two parts: (1) two 4-day low-dose training sessions in basic emergency obstetric and newborn care including newborn resuscitation ( Box 1) and (2) frequent practice during and after the training ( Figure 1). Experienced Ghana Health Service doctors and midwives were prepared as master mentors (MM) to provide the low-dose training to healthcare workers in facilities in their regions. At every facility, at least two healthcare workers were selected to become peer practice coordinators (PPCs) and were trained on the use of anatomic models and on coordinating high-frequency practice sessions to help build clinical proficiency among their colleagues. Facilitated by the PPCs, the healthcare workers practiced the skills they learned for one year during and after the low-dose trainings. In addition, all healthcare workers received mobile mentoring (mMentoring), which consisted of weekly SMS reminder messages and quiz questions on the topics covered in training. The PPCs received structured, weekly half-hour telephone calls from the master mentors, during which the mentors answered questions, provided guidance, and reinforced key messages. Healthcare workers, PPCs, and MMs received no additional incentive to attend trainings, engage in mentoring, or practice. Within one year of the initiation of LDHF, risks of intrapartum stillbirth and newborn mortality within 24 hours of birth reduced by over 50% ^12^.

**Box 1. Low dose sessions**

**Low dose session 1:**

Respectful maternity care

Infection prevention

Clinical decision-making

Support of normal labor and birth including partograph and active management of 3 ^rd^ stage of labor

Immediate newborn care

Newborn resuscitation

**Low dose session 2**

Antenatal corticosteroids for premature labor

Management of severe pre-eclampsia/eclampsia

Management of postpartum hemorrhage and repair of lacerations

Prevention and treatment of maternal and newborn sepsis

This study sought to explore healthcare workers’ experiences with this approach to in-service training, and to understand the factors contributing to intervention effects. It was one component of a mixed-methods evaluation of the Bill and Melinda Gates Foundation-funded “Accelerating Newborn Survival in Ghana” project; intervention cost-effectiveness and impacts on risk of intrapartum stillbirth and early neonatal mortality are reported elsewhere ^11, 12^.

## Methods

### Study design

In this cross-sectional qualitative study, we conducted focus group discussions (FGDs) and in-depth interviews (IDIs) at nine of the 40 health facilities included in the cluster-randomized trial to evaluate project impact ^11^. Each FGD included four to six healthcare workers who participated in the program, including doctors, midwives, and nurses providing labor, birth, and postnatal care. A total of 20 IDIs were held with PPCs and MMs. Data were collected in April 2016, at the end of the second year of the three-year LDHF program implementation. Interview guides are provided as *Extended data* ^13^.

### Sampling and study population

Study sample size was determined using qualitative criterion-based strategies, with the aim of capturing a range of perspectives reflecting the diversity of program participants. A purposive sampling technique was used to select one high-, one average-, and one low-performing health facility from each of the first three implementation waves of the LDHF program, based on the number of practice sessions held and the reduction in newborn mortality observed after the low-dose trainings. If a facility was in the top third of facilities for both indicators, they were considered high performing; facilities in the bottom third for both indicators were considered low performing. From within facilities eligible, we purposively sampled a range of large and small, urban and rural facilities to gather a variety of experiences. From within the sampled facilities, convenience sampling was used to select nine PPCs for IDIs. Nine master mentors were selected for IDIs based on convenience sampling, three from each region and two from the regional health management team. All healthcare workers who participated in the LDHF training and who were not PPCs or MMs were invited to participate in FGDs. PPCs were excluded from the FGDs to allow open dialogue about their role within the facility. All participants were over the age of 18, had participated in the implementation of the LDHF program, and provided written informed consent.

### Recruitment

Study team members sent letters to the regional health management teams to inform them of the study purpose, methods, eligible participants, and proposed dates. The regional health management teams sent letters with the same information to managers at the selected facilities. Facility management then set up appointments for the IDIs and FGDs with selected staff at the facilities. Study team members made follow-up phone calls to facilities to confirm the dates for the FGDs. The study team contacted eligible MMs by phone, explained the purpose of the study and the methods, and invited them to participate.

### Data collection

IDIs and FGDs were conducted in private rooms in the hospital maternity ward or in a conference room. All rooms afforded auditory privacy, and no non-participants were present. The study team explained the purpose, benefits, and risks of the study and obtained informed consent. Data were collected by two independent, qualitative researchers who had completed two days of training on the LDHF program and approach, informed consent and ethical principles, and qualitative methods and data collection tools. The researchers had no known prior relationship with the participants.

FGD and IDI guides were developed and pilot-tested in three hospitals outside the study regions where the LDHF training had also been conducted. IDIs lasted 40 to 60 minutes and FGDs lasted 70 to 90 minutes. All interviews and FGDs were conducted in English and audio-recorded. In each IDI or FGD, one researcher facilitated the discussion, while the other took notes. Following each session, both researchers wrote field notes on group discussion themes and other observations. Each day after data collection, the in-country principal investigator listened to the audio recordings and provided guidance to the researchers on areas they should probe further.

While the study team discussed the concept of saturation, data collectors completed the number of interviews and FGDs assigned, to ensure experiences of program participants at a range of large, small, urban and rural facilities were considered.

### Data analysis

The researchers who conducted the IDIs and FGDs transcribed all of the audio recordings. Data analysis was guided by an objectivist epistemology ^14^. Three other study team members were responsible for coding and analyzing the transcriptions, and a larger LDHF study team validated the findings. Responses were first grouped based on pre-defined codes. Deductive qualitative thematic content analysis methods were used to develop a preliminary coding scheme. Inductive methods were then used to identify emerging themes within each code. Themes and codes were reviewed and discussed throughout the analytic process, and refined or adapted as needed based on emergent information from the transcribed data. Data were analyzed by type of participant, then discussed and summarized, with differences between types of participants noted. Study team members recorded summaries of all codes individually, and then further summarized them following team discussion in order to incorporate the perspectives of all participants. Coding and analysis of themes was conducted using Atlas.ti Version 7.5 (Atlast.ti GmbH, Berlin, Germany, 2016).

### Ethical approval

Ethical approval and oversight was provided by Johns Hopkins Bloomberg School of Public Health Institutional Review Board (IRB# 5442), Baltimore, MD, USA, and the Ghana Health Service Ethics Review Committee (ERC# GHS-ERC-10/11/13) in Accra, Ghana. The study was nested within a cluster randomized trial registered in ClinicalTrials.gov ( NCT03290924).

## Results

In total, 71 individuals participated in the IDIs and FGDs (20 in IDIs; 51 in FGDs; see Table 1 for respondent characteristics). No participants dropped out of the study. What emerged from the analysis was the differences between LDHF approach and previous experiences, and the identification of relationships and motivation as enabling factors. Within each of these two themes, sub themes provide a summary of the participants’ experiences and insights.

**Table 1.** Qualitative study participant characteristics.

Characteristics	Response	Frequency, n (%)
Age, years	20–29	13 (18%)
	30–39	15 (21%)
	40–49	19 (27%)
	50+	24 (34%)
Sex	Male	2 (4%)
	Female	69 (96%)
Current Position	Obstetrician	1 (2%)
	Medical Officer	1 (2%)
	Midwife	65 (88%)
	Management	1 (2%)
	Clinical Supervision	1 (2%)
	Other (i.e. Pediatric Nurse, & General Nurse)	2 (4%)
Years of Experience (Current Position)	0 to 5	29 (41%)
	6 to 10	11 (15%)
	11 to 15	9 (13%)
	16 to 20	5 (7%)
	> 20	17 (24%)
**TOTAL**		**71**

### 1. The LDHF Approach: “It builds you your confidence”

Participants acknowledged that the content of the training was not new but how the learning experience was designed and implemented was completely different from their past experiences.

**Facility-based, team learning: *“we are all there”***

The majority of respondents noted benefits of the training being held within their own facility where all relevant colleagues could participate, democratizing the learning opportunity.

*“Even the person working can even come in, go back to work. This is not a time like somebody is choosing somebody, favoritism -- we are all there. Everybody is learning so there will be checks and balance here and there.” [MM 3]*

Healthcare workers could also balance the training with their routine work on the ward. MMs repeated sections of the training throughout the day and into the evening to ensure that all participants received training on all modules while they managed their normal duties. This reduced time away from their work and ensured that they could quickly return to the ward to attend to cases if needed. This also meant that participants with special needs (e.g. breastfeeding) could also participate. Both MMs and healthcare workers felt that the whole-team focus of the LDHF approach built a team ethos, making it easier to translate the learning to practice because everyone was trained in the same content areas and skills. This even extended to new staff or rotating students joined the facility.

“ *Oh, the experience was great. As in all of us coming together and then whatever we have, we can impart it … the students who come on rotation we gather them and then impart (lead them in practice sessions) knowledge and skill in them”. [PPC 5]*

The involvement of hospital management in the learning experience was critical. In some hospitals, managers recognized the value of the training approach and provided support during and after the training to encourage healthcare workers to participate. Participants shared examples of matrons and managers observing training and developing rosters to support healthcare workers to maintain their practice on models with positive effect on performance of healthcare workers. Participants acknowledged that without management staffs’ active participation and leadership, dedicating time to practice and introducing new procedures in the provision of clinical care would not be implemented.

*“For the facility health management team I would wish if the Medical Superintendent of this institution could also get involved; you know, if the [Nursing Director] is involved, the unit head is also involved and the Medical Superintendent also come on board, I mean, then it is going to be some form of team work …. I think people are more likely to take it [more] serious than it has been so far.” [MM 6]*

**Hands-on learning: *“then we’ll do”***

Respondents appreciated that the LDHF training approach was practical, unlike traditional theory-oriented training approaches, noting the use and benefit of birth and newborn simulators (i.e., MamaNatalie® and NeoNatalie™) during the training sessions. The skills-based approach focusing on competence of all team members, enabled them to practice and become confident in the lifesaving skills they needed on a regular basis and during emergencies.

*You will do this, you will do this because it’s a team work so during the performance of the procedure, others will be observers but one will be a leader and others will be performing the procedure. We take somebody to be the client and others will observe. After that, we assess ourselves. We assess what we have done then we give feedback to those…we ask questions to those who were observing: “What did you observe? What did you see? What have you learned? Or what is strange about the whole thing? Sometimes we try to create fun in between so that after that we share our experiences and others learn from what others do.” [FGD 5]*

During the on-site sessions MMs were able to address the needs of individual learners. For example, when facility staff performed a procedure incorrectly, the master mentors took time, one-on-one as necessary, to ensure that they were able to perform it successfully and confidently before the end of the training session.

*“You’ll read [the procedure checklist], then we’ll do. After that, you’ll do without the person reading the checklist again. Then after that we will commend the good ones, what the person was able to do right and what she [was] not able to do right; then we correct her and re-do it. Then the next person will also do it.” [FGD 12]*

Hands on practice with the models built confidence in the ability to use these lifesaving skills on real patients. Feeling confident in their skills was in of itself was also rewarding and motivating for participants.

*“What I do tell them is that it is good to come and practice on the dummy because when you do it, when you are met with a case or a problem you are able to handle it without fears, it builds your confidence. So it is not a place that we are coming to mark your grammar or any other thing but you putting your hand into practice. So when they come and do especially resuscitation and the chest is rising and falling then they are happy and excited that they have done something.” [PPC 55]*

MMs found that despite the approach yielding positive results with most participants, they still had participants who were resistant to the learning experience or struggled to achieve competence. MMs noted that they had to spend individual time with these participants and it often took much longer for them to achieve competence because they needed to first overcome the resistance to the new practices and then build their confidence to implement them.

It was evident that how MMs interacted with learners during learning sessions and demonstration on models was incredibly important. If they were supportive and managed mistakes constructively it facilitated learning and encouraged healthcare workers to learn and practice new skills. However, if MMs embarrassed the learners, particularly in front of their peers, this was demotivating, undermined confidence and in some cases, learners excluded themselves.

*You know although but we are human beings, you can make mistakes. So some dropped out…because they felt the facilitators made them feel they don’t know anything but they have some years of experiences and they think they are good. But the facilitators made them felt bad…in front of their peers so they dropped out.” [FGD_55]*

Some of the MMs and stakeholders shared that although the models were effective learning tools, only practicing on the models was limiting; they described having support while. providing direct client care as an important “final step”. Healthcare workers also noted that both time and space to were constraints to maintaining practice on the models. MMs noted that this varied among facilities, resulting in some healthcare workers being able to practice more than others. However, across all facilities, healthcare workers noted that over time the frequency of practice sessions decreased.

**mMentoring: “it reminds you”, “they call me”**

mMentoring included primarily SMS reminders and phone calls between mentors and healthcare workers. The aim of this was to provide reminders of key content covered in the training and support communication between MMs and healthcare workers. Respondents valued receiving SMS messages and quiz questions that commenced after the first learning session. They noted that the SMS messages reinforced content learned during the training sessions. The liked being able to refer to these key messages on their phones not only to remember what they learned and prompt new learning but also used as a reference guide when providing care in labor and delivery or even stimulated group discussion.

*“Sometimes I will be on the ward, [and] once I see that this labor is prolonging, I will just pick [up] my phone and open the SMS reminder and read what I should do during that prolonged labor, and after the delivery…It tells you, prepare this thing, give this thing, immediately [when] the baby is down [delivered], do this thing.” [FGD 57]*

Despite this benefit, the SMS and quizzes proved difficult for some participants and became a significant area of frustration. They shared examples of challenges with this component including difficulty interacting with the automated program, inconsistency of the messages, challenges with responding to the quiz questions, cost of responding to an SMS; all contributing to some participants deciding not to participate in the SMS and quiz component of LDHF.

The purpose of the phone calls were to enable MMs to virtually follow up with each facility after the onsite learning sessions and to encourage them to continue their practice sessions and to provide an opportunity for them to talk through any issues that they were having and to hold them accountable for doing the things that they agreed to. Mentors also provided their telephone numbers to staff and encouraged them to call if they had questions.

*Then it’s also for me—I can see that it has helped them even join the quality improvement team. So if you call them and they've not joined it gives them a reminder that they should actually join so that they know what is the way forward and how best they can help their wards.” [MM 3]*

Despite the positive feedback from all of the participants on the mMentoring component, some voiced concerns that mMentoring on its own was not sufficient for post-training follow-up and implementation support. A few of the MMs noted that they found the calls to facilities difficult to maintain. Staff would not answer their calls or did not feel that they were being honest. MMs recommended in-person mentoring for a number of reasons, in particular, to identify discrepancies between what is reported and what is actually done, and to provide direct, hands on mentoring support to healthcare workers to address gaps and solve problems. They suggested that an integrated approach including both in-person and mMentoring would be stronger.

*“Well it will be better for us to visit the facilities ourselves because sometimes you cannot really guarantee if the information you are being giving is accurate [okay]. So I think that the best thing is to visit them and see things for yourself.” [MM 6]*

### 2. Enabling implementation: “you've done well”

As participants shared and described their experiences with LDHF training they also articulated some of the factors that they felt supported implementation of what was learned during the onsite learning sessions.

**Mentorship: “together, we can solve it”**

MMs were carefully selected from each region by their regional directors. They were respected, competent and active healthcare workers themselves. The MMs themselves saw their role as being more than a traditional trainer who was responsible for imparting knowledge.

*“If there are questions, I say [the PPCs] should come and see me so that together, we can solve it, or with the participants too, those in the ward, when you take them through, if they are having any challenges too, they come and see me and we all talk about it.” [MM 8]*

They felt that they were firstly role models for healthcare workers and they had a joint vision to improve care and reduce maternal and newborn deaths. They encouraged healthcare workers to share that vision for making a difference. A key element of this was advocacy to ensure that this was a priority for management and healthcare workers alike.

*“So to enhance the mentoring, involve the people and then you have to find a way of making them add it to their top priorities that yes, this thing is that important. If you feel something is important to you, you attach all the seriousness and then allocate more time to it.” [MM 5]*

MMs initiated their relationships with teams of healthcare workers at facilities by leading the learning sessions, and maintained these relationships with teams over the next twelve months, primarily through mMentoring calls. Mentors described that a key part of their responsibility in this ongoing relationship was to provide support to health workers. This support was focused on encouraging teams to keep implementing what they had learned and to continue their practice on models. Some of the healthcare workers formed informal messaging groups with their MMs (i.e. established through WhatsApp ^TM^ messaging application) and these groups provided the platform and opportunity to solve problems and share experiences with each other. These interactions were encouraging and reinforced both learning and application of learning in patient care.

The quality of the relationship between the MM and the healthcare workers appeared to be an important aspect of the ability to provide support. If healthcare workers felt comfortable in their relationship with a MM, they would call and request assistance or discuss the problem.

*“Aside that sometimes you call them and you just encourage them. … I do not ask them about what they are doing but I just encourage them that they should encourage their colleagues to be practicing more on the Mama Natalie and Neo Natalie.” [MM 4]*
*“And then when they also have any problems. They don’t hesitate in calling me. Yes, so I think we relate well.” [MM 9]*

During these phone calls, MMs appeared to actively try to identify any problems that the team was facing and then they tried to assist teams to solve these problems. In many cases this was barriers to implementing new practices and problems that teams were encountering. Teams were encouraged to follow up request assistance from the MMs. Health care workers also provided examples of MMs directly helping teams solve problems. For example, one team worked with the Quality Improvement Team to address a commodity supply issue while another provided an example of improving data accuracy. Problem solving appeared to be collaborative, team-based and solution oriented.

*“There's a big difference because this one you have empowered the person. You have empowered them, impacted knowledge on them and then in turn they call you about their problems. You also call about their problems just to make sure that the empowerment is being used appropriately so I think this is better.” [MM 1]*

Through their ongoing supportive relationship MMs were also holding teams accountable for implementing what they had learned and what they had agreed to implement or change in their facility. Again, involvement of management was important. This strengthened the feeling of accountability. Participants noted discussing health facility results and considering what was contributing to improvements or declines and then submitting them to management for review.

*“So when you look at the information you can see whatever goes on the whole month, so you can see that this thing was high to you have to do something about it and then if fresh stillbirth is high you have to do something about it. If fresh stillbirth is high you have to discuss it, why is it going up, is it that the monitoring was not good or what. When at the end of the month I send the report, I send the report to the Medical Superintendent before sending it to the [health information officer], he will look at it. And then most of the time we have case conference, so when he sees something he will ask us why or will want to know why it is like that. I have to send the delivery books to him and he will go through it and see if something has to be done.” [PPC 13]*

**Motivating achievements: *“there’ve been changes”***

Another enabling factor that both MMs and healthcare workers described was motivation stemming from the results that they achieved. MMs described a sense of personal achievement that they got from their work. They were proud of the healthcare workers that they had worked with and appreciated the improvement that they had not only witnessed, but been part of. The immense satisfaction together with acknowledgement and appreciation of their role was a critical motivator to continue in this role. They also acknowledged that they had grown both personally and professionally and developed through the experience.

*“As in training others and then bringing them at par to where you are I believe it, it brings some joy when you realize that you've been able to tell somebody something that he or she didn't know and now through you the person knows about this and then wherever he goes and whenever he does it will either mention your name or remember you for it. So it brings some inner joy to me and that's why I always want to be a master mentor if the opportunity is there.” [MM 5]*

Healthcare workers reflected with real pride on their improved confidence and ability to provide lifesaving care. They shared examples of difficult cases and reflected on their ability to manage such cases before and after the LDHF approach was introduced. They also shared examples where they felt confident to stand up to other members of the team and provide care in the way that they thought was needed. Midwives in particular felt that a direct effect of their improved confidence and management was that fewer women needed to be referred to higher-level facilities or required assistance from doctors. They were able to manage more complicated cases independently as a result of their skills.

*“There’ve been changes. I remember there was a case, we went to theater, severely asphyxiated and when the doctor removed the baby, he said: “oh, the baby is gone.” The anesthetist came to try to suck, use the suction machine to suck and, the baby was very flabby and I mean, there was no sign of any life. So they asked me to go and bring blood from lab. And I said the baby is asphyxiated, I will resuscitate it. They said no the baby is dead, leave it and go and bring blood so that we save the mother. I went and came back and something told me that, how can I monitor this woman from morning while the baby is dead….so I went and tried to resuscitate and the baby survived. So that one alone was, I saw there’d been changes because initially I wouldn’t have been able to it and I didn’t even know how to do it. But through training, I was able to save such a baby and I was really proud of it.” [FGD_12]*

Both MMs and healthcare workers noted a significant change in the way that care was provided and how women and their families were treated. The training included a component of “respectful maternity care” and participants shared that this concept was new for them but in practice, it has made a big difference to their clients how their facility is perceived in the community. Respectful maternity care included giving clients and their companions the opportunity to understand and make decisions about the procedures performed during labor, birth, and the postnatal periods, such as examinations being performed and the position in which they prefer to give birth. As a result, clients were more cooperative and appreciative of the care they received, and even referred friends to the facility to give birth.

*“Because of the nice way and the quality care we are giving to them … people will come from maybe Accra or any place. When you ask “Why have you come?” They will say a sister told them we provide quality services here at our facility so I'm coming to deliver here. So for this matter, it seems our message is going far or our services are going far and people are coming, but it wasn’t that before the training.” [FGD_13]*

For participants, the most motivating of their achievements was that as a direct result of their improved management, confidence and skills—more mothers and babies were getting the care that they needed, being referred less and having better outcomes. Nearly all participants felt that there was a reduction in maternal and newborn mortality in their facilities because of the improved care that women were receiving. There was a feeling of ownership, purpose and pride that their improved skills and practice was having this tangible impact.

*“Our stillbirths have reduced drastically and then neonatal deaths too have reduced. Following the skin to skin it has also made that one to reduce. Now our doctors also have some trust in us. When we are able to manage a case, afterwards you call him and give him the feedback. He will say okay”. [FGD_30]*

## Discussion

Quality care is a priority for all countries to realize their commitment to Universal Health Coverage in the Sustainable Development Goals era ^15^. However, quality of care is low in many countries, and research has demonstrated that healthcare workers typically provide less than half of the evidence based interventions expected for certain health needs ^16^. A combination of training and supportive supervision has been the most prominent intervention to improve healthcare worker performance and assure quality care. However, as Leslie *et al*. argue, these interventions have fallen short and have not meaningfully improved the performance of healthcare workers nor quality of care ^17^. Similarly, the systematic review by Rowe *et al*. on approaches to improve healthcare workers’ performance found great variability among interventions. Although training and supportive supervision was a common combination, it still yielded modest improvements and varied between settings. Based on this, review recommendations included trying to identify and understand attributes of effective strategies (especially training and supervision), cost and context in order to design more effective interventions ^5^.

This study complements impact and cost-effectiveness evaluations of this LDHF training by providing insights into the experiences of direct beneficiaries. Incremental cost and cost-effectiveness analysis showed that the LDHF approach, with onsite training, peer practice, a low-cost mobile messaging system, and mentorship phone calls, demonstrated good value for money and significant reduction in both stillbirths and immediate newborn deaths ^11, 12^. Together, these findings suggest that the LDHF approach is a practical and cost-effective strategy for healthcare worker capacity building in Ghana and similar settings. A concurrent study of a similar facility-based, team training intervention for prevention and management of postpartum hemorrhage and birth asphyxia in Uganda found similar results. Intrapartum stillbirths decreased by 34% and early neonatal deaths decreased by 62% from baseline to endline, and remained reduced for 6–9 months post intervention ^18^. Interviews with facility staff and district trainers suggested that facility staff who practiced more were motivated by a desire to maintain skills and be prepared for emergencies, external recognition, and establishing a set schedule for practice ^19^.

These insights help us understand how and why the LDHF approach affected healthcare workers’ performance and achieved the results that it did. The LDHF approach was more than a training event for participants. It built skills and confidence to practice. The positive relationship established between healthcare workers and MMs and contribution to meaningful results appeared to motivate further improvement and a commitment to maintaining achievements.

The findings are consistent with findings by Niles *et al*., suggesting that instructor-led, onsite training combined with regular feedback improves knowledge and competency and promotes retention ^20^. In addition, Wulf *et al*. and Bosse *et al*. showed that both low- and high-frequency intermittent positive feedback, as compared to continuous concurrent (permanent) feedback, leave sufficient time for self-directed practice, which leads to skill retention ^21, 22^. This peer mentoring approach aligns with WHO’s position that experienced members of the clinical team can lead continuing training activities such as meetings or morbidity and mortality rounds ^9^.

Quality improvement researchers have highlighted the need to understand how contextual factors influence improvement efforts. In this study, participant reflections support Ovretveit’s notions that quality improvement interventions are an “inter-dependent” set of activities that result in many types of changes (e.g., increased confidence, respectful care, implementing new skills) ^23^. These many changes together improved the outcomes of mothers and newborns on the day of birth. Through understanding their experience and insights of the intervention participants, we might understand what components of the intervention were the most important.

This study does have limitations. First, due to the cross-sectional design and limitation of the sample to health workers who were involved in the LDHF program, we cannot generalize findings to all hospitals. However, the study sought responses from high- and low-performing, and urban and rural hospitals. Some participants may have been hesitant to share negative experiences about the LDHF approach, but we believe the use of independent qualitative researchers to collect all data minimized sponsor bias. Finally, the analysis did not include respondent validation of the results.

Damschroder *et al*. ^24^ argue that we need to understand why an intervention works, including “what factors influence implementation and how implementation influences performance of the intervention.” They propose five domains to explore: intervention characteristics, outer setting, inner setting, characteristics of the individuals involved and the process of implementation ^24^. Further exploration of these domains will help us understand why interventions work and why replication of a successful intervention might not achieve the same results in different environments ^25^. Future studies should evaluate the LDHF approach in other technical areas and settings, to understand what types of learning needs benefit most from this multi-faceted capacity building approach and how context influences intervention effectiveness. Adaptations of the approach, such as including facility management and training managers in the program, modifying intensity of intervention components, and scaling down to lower levels of the health system with smaller caseloads, should also be further evaluated.

## Conclusions

Reflections from participants of a low-dose, high-frequency training program to accelerate reductions in newborn mortality in Ghana reinforce emerging evidence on the characteristics of effective strategies to improve healthcare worker performance and quality of health services in low- and middle-income countries. Healthcare workers felt that a skills-based training approach, hands on practice, and mMentoring strengthened their technical capacity and confidence, facilitated translation of skills into routine service delivery, and improved the quality of the maternal and newborn services they provided. Further research is needed in other settings to understand how context can influence healthcare worker experiences and the effectiveness of multi-faceted capacity building approaches such as this one.

## Data availability

### Underlying data

Qualitative data files may contain text that directly or indirectly identifies study participants. Data can be made available for further analysis upon reasonable request and signature of a formal data sharing and use agreement, provided research objectives are aligned with original study aims. Requests may be submitted to IRBHelp@jhpiego.org.

### Extended data

Figshare: Accelerating Newborn Survival in Ghana_ Qualitative Data Collection Tools. https://doi.org/10.6084/m9.figshare.8150678.v1 ^13^.

Extended data are available under the terms of the Creative Commons Attribution 4.0 International license (CC-BY 4.0).
