# Supplementary material for: “It builds your confidence… you’ve done well”: Healthcare workers’ experiences of participating in a low-dose, high-frequency training to improve newborn survival on the day of birth in Ghana
Source: Gates Open Res. 2019 May 22;3:1470. [Version 1] doi: 10.12688/gatesopenres.12936.1 (PMC6667826; doi:10.12688/gatesopenres.12936.1)
Supplement: Peer review history

# Peer review: Reviewer response for version 1

**DOI:** 10.21956/gatesopenres.14039.r27530

**Published:** 2019-07-30

**Authors:** Romano Nkumbwa Byaruhanga

**Affiliations:** 1 Department Obstetrics and Gynaecology, St. Francis Hospital Nsambya, Kampala, Uganda

**Focus of study: **

- This is an important research focusing on strategies that are multi-pronged in nature in order to improve on implementation of evidence based interventions for quality care improvement in maternal newborn care in low resource settings.

**The Background section, justification and objectives for the study:**

- Well written and described.

 

**Study Design: **

- Suggest the authors to change the statement to reflect: A phenomenological design was used and involved FGDs and IDIs at nine of the 40 health facilities included in the cluster-randomized trial to evaluate project impact.

**Sampling, Study Population and Data analysis: **

- Thick description of the process, interviews, process, transcription and analysis. However, in the study it is not clear whether the number of FGDs and IDI were predetermined prior to the start of the study. Was the eventual number obtained in the field after theoretical data saturation? Kindly comment on this.
- It is not enough to state that the study team discussed the concept of saturation. Was saturation reached and if so when?
- There were 69 female participants presenting 96%. Were all cadres (specialists, doctors, midwives, nurses) mixed together when the FGDs were conducted?
- Were there any issues regarding gender differences, and on how responsibilities of roles in the medical hierarchical scenario were handled during the FGDs?
- Three other study team members were responsible for coding and analyzing the transcriptions, and a larger LDHF study team validated the findings. Responses were first grouped based on pre-defined codes. Please state or initial the study team members who were involved in the analysis.

**Discussion:**

- I would suggest the authors to state the two main themes derived from the study in the first paragraph. Also, to have a comment on the trustworthiness and credibility which was demonstrated during the study. There was prolonged engagement, peer debriefing, triangulation of different data collection methods (IDS and FGDs), thick description of the study process, members check and reports on negative cases.

**Methodological limitation of the study**:

- Only PPC and MMs were interviewed about the LDHP process. No other health workers in the maternity or pediatrics section were interviewed; this could create a bias as these were workers initially primed and recruited in the study and hence their opinion could be biased about the process.
- The researchers who collected and transcribed the data were not directly involved in the conceptualization of the study. The data analysis was done by the study team and LDHF study team. What was the background of these members in the study team? Kindly describe this and state if it created any bias.

I confirm that I have read this submission and believe that I have an appropriate level of expertise to confirm that it is of an acceptable scientific standard.

# Peer review: Reviewer response for version 1

**DOI:** 10.21956/gatesopenres.14039.r27234

**Published:** 2019-07-12

**Authors:** Regien Biesma-Blanco

**Affiliations:** 1 Department of Health Sciences, Global Health, University Medical Centre Groningen/University of Groningen, Groningen, The Netherlands

This is an important study exploring healthcare workers’ experiences with the LDHF approach to improve newborn survival. The study consisted of 20 IDIs with Master Mentors (MMs) and Peer Practice Coordinators and 9 FGDs with health professionals working in the facilities. Overall it was concluded that the experience with the approach LDHF was positive, both for the staff themselves and also for maternal and newborn health outcomes.

 Some negative feedback included the demeaning attitude of some MMs and that staff had not taken it that serious if management had not been involved. This is perhaps a point of critique - in that this could have been explored in more depth. In fact, most of the quotes in the paper were from MMs (who were the trainers not participating staff) and who are likely to have been more positive about the LDHF approach. This could have been made more explicit - were the any contrasting views within the FGDs (staff) or with the IDIs (trainers) and how did the authors deal with that?

 Nevertheless, this is a well written and scientifically sound paper about an important global health topic.

I confirm that I have read this submission and believe that I have an appropriate level of expertise to confirm that it is of an acceptable scientific standard.
